# The impact of green finance on the optimization of industrial structure: Evidence from China

**DOI:** 10.1371/journal.pone.0289844

**Published:** 2023-08-10

**Authors:** Xing Xiong, Yuxing Wang, Bin Liu, Wenhong He, Xinghou Yu

**Affiliations:** 1 Research Center for Economy of Upper Reaches of the Yangtze River, Chongqing Technology and Business University, Chongqing, China; 2 School of Economics, Chongqing Technology and Business University, Chongqing, China; East China Normal University, CHINA

## Abstract

Green finance promotes the optimization of industrial structure and continuous improvement of ecological environment by supporting the development of green industries. Based on the panel data of 30 provinces in China from 2012 to 2020, this paper uses the entropy weight TOPSIS method to measure the development level of green finance and the level of industrial structure optimization in China, and constructs a panel data model to empirically test the impact of green finance on the upgrading of China’s industrial structure. The study finds that there is still an imbalance and insufficiency in the development of green finance and industrial structure optimization in China. From 2012 to 2020, the development level of green finance and the level of industrial structure optimization in China have been continuously rising, but there is obvious heterogeneity, showing an eastern>central>western spatial pattern. Empirical analysis results show that at the significance level of 1‰, the development of green finance has a significant promoting effect on the rationalization and upgrading of the industrial structure. However, there is significant heterogeneity in the impact of green finance on industrial structure optimization. In terms of regional heterogeneity, at the significance level of 1‰, the role of green finance in promoting the optimization of industrial structure in central and western China is higher than that in eastern China, and the impact of green finance on China’s industrial structure shows a spatial pattern of western>central>eastern China. In terms of industry heterogeneity, at the significance level of 1‰, green finance has a significant promoting effect on the development of green industries, and a significant inhibiting effect on the development of high-energy-consuming industries. Specifically, in the green industry, green finance has the greatest promoting effect on the communication and other electronic equipment manufacturing industry; in the high-energy-consuming industry, green finance has the greatest inhibiting effect on the black metal smelting and rolling processing industry, and the smallest impact on the petroleum, coal and other fuel processing industry. Finally, based on this, policy suggestions for green finance to support the optimization of industrial structure are proposed from two dimensions: government and financial institutions.

## 1. Introduction

Industrial development has always been the basic force that drives economic and social advancement, and industrial structure changes are often an important reflection of economic and social development. However, the industrial structure itself needs to be continuously optimized and improved to meet the economic and social development [1]. In order to support environmental protection and cope with climate change, green development has become the trend and direction of industrial structure optimization [2]. Green finance can play an important role in promoting the transformation and upgrading of industrial structure towards a more sustainable path. By reducing the financing cost of green enterprises and increasing their competitiveness, green finance can encourage investment in green projects and technologies, and promote environmental protection and governance [3]. At the same time, by increasing the financing cost of polluting enterprises and limiting their access to credit, green finance can create incentives for these enterprises to adopt cleaner production processes and technologies, or exit the market. By channeling capital towards green and sustainable industries, green finance can promote the optimization and upgrading of industrial structure, while reducing the negative environmental impacts of economic development. Overall, green finance can be a powerful tool for guiding the allocation of financial resources towards a more sustainable and inclusive economic development path.

In recent years, China’s green financial system has been gradually improved and the role of green finance in promoting the green transformation of China’s industrial structure has become increasingly prominent. According to China’s National Bureau of Statistics, by the end of 2020, the balance of green loans in China was about US$1.8 trillion and the stock of green bonds was about US$125 billion. However, the current development of green finance in China is still facing practical problems such as insufficient scale, mixed flows and vague concepts. In terms of the supply of funds for green finance, the expansion of green credit in China still lacks direct and effective support and incentives, resulting in a still low proportion of green credit in the total scale of all types of credit. By the end of 2020, green credit in China will only account for 6.88% of the total credit scale.

**Fig 1:**
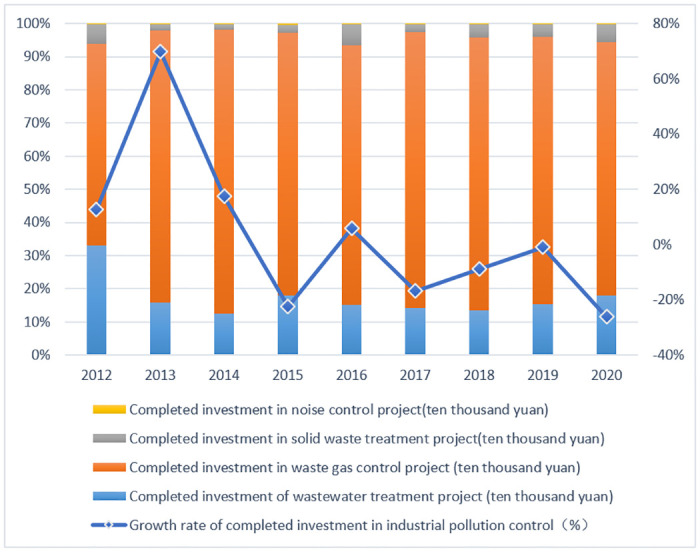
Scale of investment in pollution control in China, 2012–2020.

In terms of green investment, a 2015 Chinese central bank report showed that to reach the pollution reduction targets established by the Ministry of Environmental Protection, the government would need to make an annual investment of 2 trillion yuan ($320 billion), with the government budget covering only 15 percent of the total investment. Fig 1 shows that from 2012 to 2020, the total amount of pollution control investment completed each year is still less than 100 billion yuan, and has not formed a growth trend. This indicates that the current supply of funds for green finance can hardly support the rapid growth of green investment demand.

In terms of the composition of projects supported by green finance, green credit and green bonds, which account for the highest proportion of green finance, have problems such as mixed flow of funds, which reduces the green benefits of green financial development. For example, the total amount of green bonds issued in China in 2017 was US$37.1 billion, ranking second in the world after the United States, but 38% of them did not meet the international definition of green bonds, and some of the funds raised were used for projects such as general business operations and fossil fuel assets, which instead further aggravated the current serious ecological and environmental problems.

From the data published by the CBRC, the highest percentage of green credit flows mainly to railroad transportation projects and urban transportation projects, which together accounted for 40–50% of the total green credit in the calendar year. Which deviates to a certain extent from the concept of green credit that mainly covers green projects such as new energy development, energy-saving and environmental technology advancement and ecological environmental protection. Thus, it may weaken the role of green credit in supporting the optimization of industrial structure.

With the worsening ecological environment problems, accelerating the construction of a green financial system, increasing the effective supply of green funds, and clarifying the path of green finance to promote the transformation and upgrading of industrial structure have become important issues for scholars and governments worldwide. This paper contributes to this field in several ways. Firstly, it confirms the significant positive effect of green finance on China’s industrial structure transformation and upgrading, providing theoretical supplements and empirical evidence for relevant studies on innovative government resource allocation, improving the efficiency of financial resource allocation, and promoting green development and industrial transformation. Secondly, it distinguishes itself from existing studies by using a green finance evaluation index system measured by a combination of several factors to study the typical facts of green finance and industrial structure optimization in China, providing a new comprehensive measure of green finance. Thirdly, it proposes new insights on the green transformation of industrial structure by studying the direct and indirect impact paths of green finance on the transformation and upgrading of industrial structure, taking green enterprises and polluting enterprises as the research objects respectively, based on the connotative qualities of green finance.

## 2. Literature review

In the face of global climate change, regional pollution and energy depletion, the United Nations first put forward the concept of "green" at an international public forum in the 1980s, and called for the establishment of a "green" industrial system to achieve sustainable economic growth. With the in-depth understanding of economic activities, energy consumption and pollution emissions, how to solve the problems of economic growth and ecological sustainability has become one of the focuses of economic research. Therefore, further research paradigms are proposed, such as green growth, green transformation and green development [4–7].

With the gradual strengthening of public awareness of environmental protection and environmental enforcement standards, environmental regulations are becoming more and more constraining to industrial restructuring. Promoting the ecological development of industrial structure has become one of the important ways to achieve green and low-carbon development [8]. How to promote the ecological transformation of industrial structure, reduce the consumption of fossil energy such as coal, and reduce the intensity of carbon dioxide emissions is an important issue that needs to be urgently addressed in response to global climate change [9].

The existing literature has three main views on the development of industrial structure in the context of environmental regulation. (1) The "cost compliance theory" argues that enhanced environmental regulation will lead to increased production costs, which in turn will inhibit technological innovation and thus will weaken the competitiveness of firms [10]. (2) The "innovation compensation theory" argues that reasonable environmental regulation can stimulate firms’ technological innovation and promote the optimal allocation of resources, which will offset the cost of environmental compliance, thus improving industrial competitiveness and achieving a win-win situation in which environmental performance and economic performance are simultaneously improved [11]. (3) Some other scholars believe that the impact of environmental regulation on industrial structure depends on the size of the "innovation compensation effect" and the "compliance cost effect", showing typical nonlinear characteristics [12].

Industrial development is the core of the economic system, which not only affects the degree of utilization and efficiency level of various production factors in the economic system, but also directly determines the type and scale of pollution emission in the process of economic operation. Industrial structure optimization includes industrial structure rationalization and advanced, and industrial structure optimization is a dynamic process of spiral [13]. From the research history of industrial structure optimization, we can analyze the connotation of industrial structure optimization from macroscopic perspective and microscopic perspective.

From the macro dimension, industrial structure optimization is more concerned with the industrial structure change and the integration with the value chain theory, reflecting the overall industrial structure optimization of society through the change of the share of each industry. Dogan and Inglesi (2020) specify the basic pattern of industrial structure optimization in the process of economic development, that is, as the economy develops, the output and labor share of the agricultural sector gradually decreases, the share of the industrial sector rises and then falls, and the share of the service sector rises slowly and then becomes rapidly increasing [14]. With the emergence of value chain theory, scholars further researched industrial structure optimization. in the 1990s, Gereffi (1994) proposed the concept of "global commodity chain" and focused on the internal structural relationship of global commodity chain, which opened up the research on industrial structure optimization under the idea of value chain [15]. They further analyzed the theory and framework of global value chain, emphasized the importance of value creation and value acquisition in each link of the value chain, and believed that industrial structure optimization is directly expressed as the process of gradual upgrading of an enterprise, a region or a country along the value ladder in the global value chain.

As research progressed, scholars began to focus on the micro factor of production level. The issue of industrial structure upgrading is studied at the micro-firm level, and it is argued that the shift of firms to capital- and technology-intensive industries drives industrial upgrading. Wang X et al (2018) proposed that upgrading of manufacturing firms is the process of transforming firms from producing labor-intensive products to producing capital- and technology-intensive products [16]. Ding Huanfeng et al (2018) found that technological progress will increase the productivity of enterprises, and factors of production will therefore flow to the sector, promoting the development and upgrading of the industry [17]. Upgrading the industrial structure also involves continuous participation in more valuable business activities, producing more high value-added products or participating in high value-added activities in order to improve the relative competitiveness in the value chain. In addition, they are able to create stronger ties with core companies within the supply chain and give them better potential revenue. To sum up, at the macro level, industrial structure optimization refers to the tilting of industrial structure toward the tertiary industry and the continuous improvement of industrial status in the global value chain. At the micro level, industrial upgrading refers to enterprises producing high value-added products, enhancing product competitiveness and reaping more benefits through continuous innovation and technological progress.

As an important tool for capital allocation, finance plays a supporting role in optimizing industrial structure. It is expressed in the formation of mechanisms to promote technological innovation, the growth of SMEs, industrial integration and industrial spatial agglomeration [18]. Financial resources are necessary for the optimization of industrial structure. Financial activities can have a significant impact on the structure of industries. There is a long-term equilibrium between financial development and industrial structure optimization, and financial development is more conducive to the increase of tertiary industry output. Guiding financial resources into the green renewable energy industry can promote the upgrading of the industrial structure to a green structure [19], thus enhancing the momentum of economic development. Chang et al. (2019) point out that the implementation of financial policies can guide the free flow of social capital between industries and provide financial security for industrial development [20]. Hua-qian and Yi-qing (2010) believes that there is a clear mutual influence and promotion relationship between financial development and industrial restructuring [21]. Hu et al.(2017) found that financial institutions optimize the efficiency of resource allocation by increasing investment in emerging industries and decreasing investment in sunset industries [22].

Globally, the green finance issue started in the 1980s when social, resource, and environmental problems were becoming increasingly serious [23]. Since the adoption of the Rio Declaration on Environment and Development and Agenda 21 at the United Nations Conference on Environment and Development in 1992, and the signing of the United Nations Framework Convention on Climate Change to address global climate change, countries around the world have been paying great attention to energy conservation and environmental protection, and green finance has been rapidly promoted as a grip for economic development under this concept. The signing of the Kyoto Protocol in 1997 marked the further promotion of green finance development in developed countries [24]. Regarding the rapidly deteriorating ecological environment, scholars believe that in order to fulfill their social obligations, companies have the responsibility to prevent pollution in their production and the financial sector has the responsibility to make biased investments. The financial sector has given birth to the concept of green finance by strengthening the financing constraints of brown and black economic activities while providing ample financial support for green economic activities. That is, enterprises have the responsibility to prevent pollution and make more green investments in their production, while the financial sector has the responsibility to guide enterprises to make green investments through the optimal integration of capital allocation. With the continuous development of green finance, scholars have begun to pay attention to the correlation between green finance and industrial structure. Cowan (1998) first analyzed the relationship between green finance and industry from the perspective of green financial instruments such as green loans, green financial bonds, green environmental protection funds, carbon dioxide emission trading and environmental pollution liability insurance [25]. They point out that green finance is a bridge between the financial industry and the environmental protection industry, aiming at guiding the flow of capital to the environmental protection industry and realizing the optimization and adjustment of the industrial structure. Mkl A (2016) argue that the purpose of developing green finance is to implement the concept of environmental protection in depth and to achieve high-quality economic development [26]. Dikau and Volz (2021) argued that green finance is different from traditional financing methods, and by creating diverse green financial instruments, it promotes the development of environmental projects, limits the advancement of polluting projects, and ultimately realizes the ecological restructuring of industries [27]. Yu et al (2021) pointed out that green finance can discourage investment in energy-intensive industries and enhance investment in technology-intensive industries, ultimately achieving the goal of industrial restructuring [28].

In summary, previous studies can provide useful references, but there are still two areas that need further enrichment and improvement. Firstly, the limited scope of empirical testing, which has mainly focused on the aggregate level of industrial structure indicators, such as the share of green industries in GDP or employment. However, there is a need for more granular empirical analyses that examine the impact of green finance on specific sub-sectors of the economy, such as energy-intensive industries, which may face different challenges and opportunities in the transition towards a greener and more sustainable industrial structure. Secondly, the measurement of green finance, which has often been limited to a single dimension, such as green credit or green bonds. However, green finance is a complex and multidimensional concept that involves various types of financial instruments, including loans, bonds, equity, and insurance, as well as different sources of finance, such as public and private sources. Therefore, a more comprehensive evaluation index system for green finance is needed to capture its various dimensions and provide a more accurate assessment of its impact on industrial structure optimization.

Based on this, three additions have been made in this paper: Firstly, at the theoretical level, the mechanism of the influence of green finance on industrial structure upgrading is theoretically analyzed, and research hypotheses are proposed. Secondly, at the level of index system, the evaluation system of green finance is constructed from four aspects: green credit, green investment, green insurance, and green fiscal expenditure. Thirdly, at the level of subdivided industries, the high-pollution and high-energy-consuming industries are refined into six major industries, namely petroleum, coal and other fuel processing industry, chemical raw materials and chemical products manufacturing industry, electric power and heat production and supply industry, non-metallic mineral products industry, ferrous metal smelting and rolling processing industry, and non-ferrous metal smelting and rolling processing industry; the green industries are specified into three major industries, namely, agricultural and sideline food processing industry, pharmaceutical manufacturing industry, and computer, communication and other electronic equipment manufacturing industry. The impact of green finance industry on different industries is analyzed by industry to verify the resource allocation effect of green finance on industrial structure upgrading.

## 3. Mechanism analysis and research hypothesis

**Fig 2:**
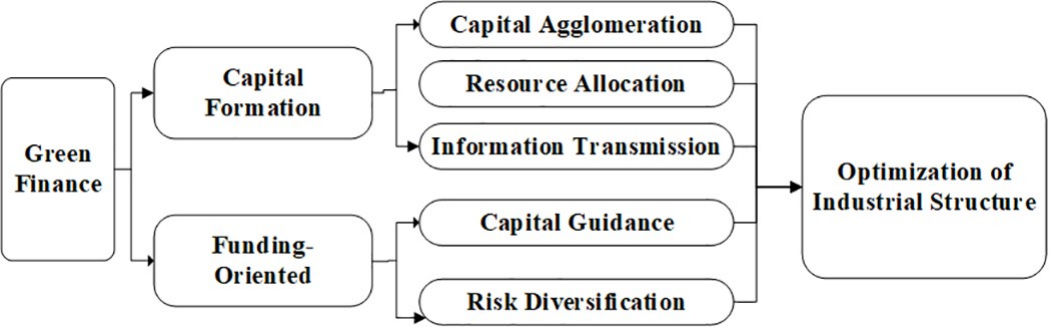
The impact path of green finance on industrial structure optimization.

The impact of green finance on industrial structure optimization is primarily reflected in the level of resource allocation. Through the effective allocation of market capital, green finance promotes the development of green and high-tech industries while inhibiting the development of high-pollution and inefficient industries [5, 29]. Compared to traditional finance, green finance is more concerned with the environmental impact of industrial economic development. Based on its fundamental attributes, green finance offers investment guidance, capital aggregation, resource integration, and information transfer functions, which can effectively facilitate the green and sustainable transformation of regional industries. As shown in Fig 2.

### 3.1 Capital formation mechanism

From the perspective of capital accumulation function, the production, operation, and transformation of enterprises cannot do without adequate financial support. As a new financial model evolved from traditional finance, green finance can achieve capital accumulation and rational allocation of green credit funds through financial product and instrument innovation [30, 31]. It can also expand the investment and financing channels of green and clean industries and provide sufficient material guarantees for regional industrial transformation. However, due to the differences in economic development levels among regions, there are differences in the development level of green finance and its impact on the upgrading of industrial structure.

Meanwhile, finance itself has the function of regulating the allocation of social resources [32]. Green finance, through managing differentiated credit rates, channels a large amount of existing funds into green and high-tech industries, while promoting the transfer of human, capital, and technological resources to clean industries. This encourages other industries to actively reform their production processes and transform their production methods, ultimately achieving a green transformation of the entire region’s industries.

In addition, according to the efficient market theory, transaction prices in the securities market can effectively reflect product information [33]. Against the backdrop of increasingly strict information disclosure, green industries have a higher investment value in the market. Green finance utilizes the transaction prices in the securities market to transmit policy signals of green development to the public, further strengthening the guiding role of credit funds in green finance and promoting the transformation and upgrading of regional industries. Green finance releases investment and financing information, reduces the cost for investors to search for and evaluate green projects, and promotes the participation of social capital in green industrial investment. At the same time, the government advocates the development of green finance through policy guidance, enabling green finance to play a resource allocation function and support the upgrading of traditional industries.

Based on this, the research hypothesis was formulated.

**Hypothesis 1**: Green finance helps to promote the rationalization of regional industrial structure.
**Hypothesis 2**: Green finance helps promote the development of advanced regional industrial structure.
**Hypothesis 3**: There is regional heterogeneity in the impact of green finance on industrial structure upgrading.

### 3.2 Funding-oriented mechanism

Green finance refers to a series of policies and institutional arrangements that guide social capital into the development of green industries such as environmental protection, energy conservation, clean energy, and transportation, through financial services such as loans, private equity funds, bond and stock issuances, and insurance. It is an important financial support for optimizing the industrial structure. Green finance, especially green credit policy tools, adjust credit interest rates, regulate innovative credit quotas, and use differential leverage to create an interest spread between green and clean industries and high-polluting industries in terms of credit costs, investment risk control, and investment returns. This encourages credit funds to flow from high-polluting industries to green and clean industries, achieving the fund guidance function of "restraining pollution and promoting green". On the one hand, green credit compresses the financing scale of heavily polluting industries by raising the financing threshold for these industries and refusing to provide loans to high-polluting enterprises. This effectively alleviates the resistance of the "two highs and one surplus" industries to regional industrial transformation and forces high-polluting, high-emission, and low-efficiency industries to undergo technological innovation [34–36]. On the other hand, green credit also actively channels funds into green and high-tech industry sectors, alleviates the financing pressure on emerging enterprises, effectively promotes the development of clean industries, and promotes regional industrial transformation and upgrading.

Accordingly, the following research hypothesis is proposed.

**Hypothesis 4**: Green finance has a disincentive effect on industries with high pollution and high energy consumption.
**Hypothesis 5**: Green finance has a promoting and supporting effect on green industries.

## 4. Data, descriptive statistics and empirical strategy

### 4.1 Data description

The empirical analysis in this paper is based on a panel dataset of 30 Chinese provinces from 2012 to 2020. The sample period for this study was set to 2012-2020. The reason is that in November 2011, China’s Ministry of Environmental Protection issued the "12th Five-Year Plan for the Construction of National Environmental Protection Regulations and Environmental and Economic Policies", which proposed to actively explore green finance. Since then, China has gradually established a new model of supporting green development through finance, and the Chinese color finance policy system has been continuously improved. Therefore, we set the sample period from 2012 to 2020, which is more helpful to analyze the impact of green finance development on industrial structure optimization.

The data in this paper are mainly from China Industrial Statistical Yearbook, China Insurance Yearbook, and China Statistical Yearbook from 2013–2021.

### 4.2 Variable description

#### 4.2.1 Green finance

*1*. *Evaluation index system*. Green finance refers to the use of green credit, green investment, green insurance and government support and other financial tools to guide the flow of funds to green industries, thereby promoting the green development of industries.

We constructed an indicator system based on the principles of systematicness, scientificness, comparability, representativeness, and operability. In terms of indicator selection, we referred to the existing literature on green finance and based it on China’s "Guiding Opinions on Building a Green Finance System," constructing a green finance evaluation indicator system from four dimensions: green credit, green investment, green insurance, and government support [37–40]. As shown in Table 1.

**Table 1:** Evaluation index system of green finance.

Indicators	Description of indicators	Indicator Properties
Green Credit	The proportion of interest expenses in high-energy-consuming industries to total interest expenses in industrial sectors	Negative indicators
Green Investment	Investment in environmental pollution control as a percentage of GDP	Positive indicators
Green Insurance	Ratio of agricultural insurance income to total agricultural output	Positive indicators
Green Fiscal Spending	Ratio of financial environmental protection expenditure to financial general budget expenditure	Positive indicators

*2*. *Evaluation method*. We use the entropy-weighted TOPSIS method to measure the level of green finance development in China.

The entropy-weight TOPSIS method combines the TOPSIS method with the entropy value method to address the issue of inconsistency in evaluation results caused by changes in the evaluation environment or conditions. By using the entropy method to determine the weight of each evaluation index, the method can effectively eliminate the influence of variable factors on the evaluation results, making the results more comparable [41]. In the TOPSIS method, the ideal and negative ideal solutions are identified, and the distance between each evaluation object and these solutions is calculated. Based on these distances, a relative closeness coefficient is calculated for each object, and the objects are ranked accordingly. However, when the evaluation environment or conditions change, the ideal and negative ideal solutions may also change, leading to inconsistent evaluation results. The entropy method is used to determine the weight of each evaluation index, taking into account the degree of dispersion of the index values. This ensures that the weight assigned to each index is proportional to its contribution to the overall evaluation [42]. By combining the TOPSIS method with the entropy method, the entropy-weight TOPSIS method provides a more robust and consistent approach to evaluation, which is especially useful in situations where the evaluation environment or conditions are subject to change.

#### 4.2.2 Industrial structure optimization

*1*. *Evaluation index system*. Industrial structure optimization is a dynamic process, through the continuous adjustment of each industry to promote coordinated economic development in order to achieve a state of high efficiency and rational resource allocation [43]. It is generally believed that industrial structure optimization includes industrial structure rationalization and industrial structure heightening. Rationalization of industrial structure refers to a dynamic process of reasonable allocation of resource factors among industries and balanced supply and demand of industrial structure under the condition of certain technology level, and the manifestation of industrial structure rationalization differs in different stages of economic development [44]. The advanced industrial structure is a process in which the industrial subject evolves from a low-level form to a high-level form [45]. According to the allotment-Clark theorem, Hoffman’s law and Kuzlitz’s law, the advanced industrial structure can be understood as the decline of the share of primary industry in the industrial structure, the increase of the share of secondary and tertiary industries, and finally the dominance of tertiary industry [46]. Based on this, this paper analyzes the optimization of China’s industrial structure from two dimensions: rationalization of industrial structure and advanced industrial structure with reference to the approach of Spracklen (2016) [47]. The specific evaluation indexes are shown in Table 2.

**Table 2:** Evaluation index system of industrial structure optimization.

Primary Indicators	Secondary Indicators	Calculation method	Indicator Properties
Industrial structure optimization	Rationalization of industrial structure	See below Eq. 4.13	Negative indicators
	Advanced industrial structure	See below Eq. 4.14	Positive indicators

**2. Evaluation methodology**. *①Rationalization of industrial structure*. The rationalization of industrial structure refers to the aggregation quality between industries. On the one hand, it reflects the degree of coordination among industries, and on the other hand, it should also reflect the degree of efficient utilization of resources. In other words, it is a measure of the coupling degree between factor input structure and output structure. As for this coupling, researchers generally use the degree of structural deviation to measure the rationalization of industrial structure, which is formulated as follows:

$$E=\sum_{i=1}^{n}\left|\frac{Y_{i}/L_{i}}{Y/L}-1\right|=\sum_{i=1}^{n}\left|\frac{Y_{i}/Y}{L_{i}/L}-1\right|$$ (1)

In the formula, E denotes the degree of structural deviation, Y denotes output, L denotes employment, i denotes industry, and n denotes the number of industrial sectors.

According to the assumptions of classical economics, the economy ends up in equilibrium with the same level of productivity in each industrial sector. By definition, Y / L means productivity, so when the economy is in equilibrium, Y_i_ / L_i_ = Y / L, and thus E = 0. Also, Y_i_ / Y denotes the output structure and L_i_ / L denotes the employment structure, so E is also a reflection of the coupling of the output and employment structures. The larger the E value, the more the economy deviates from equilibrium and the more irrational the industrial structure. Since economic disequilibrium is a common phenomenon, particularly in developing countries where it is more pronounced, it is impossible for the E value to be zero. However, the structural deviation index treats each industry equally, ignoring their respective importance in the economy. Additionally, the calculation of absolute values also poses inconveniences for research. Therefore, in this study, following the approach of Gu R et al (2022) [48], the Theil index is used to evaluate the level of rationalization of the industrial structure. Its calculation formula is as follows.


R=∑i=1nYiYlnYiLiYL (2)

Similarly, if the economy is in a state of equilibrium, R = 0. The index takes into account the relative importance of industries and avoids the calculation of absolute values. At the same time, it retains the theoretical basis and economic significance of structural deviation, making it a better measure of industrial structure rationalization. When the Theil index is not equal to zero, it indicates that the industrial structure deviates from the equilibrium state and is unreasonable. The larger the R value, the greater the degree of deviation of the industrial structure, the poorer the efficiency of the industrial structure, and the more unreasonable the industrial and employment structures are. The closer the R value is to zero, the smaller the degree of deviation of the industrial structure, the better the efficiency of the industrial structure, the more reasonable the industrial and employment structures are, and the more mature the industry is.

*②Advanced industrial structure*. The upgrading of industrial structure refers to the development process of industry structure from low-level to high-level, from simple to complex products, and from low-end to high-end value chains. Currently, the main indicators for measuring the upgrading of industrial structure include the Moore value, K value, and the coefficient of industrial structure hierarchy. In this study, the selection and measurement of indicators for the rationalization of industrial structure mainly refer to the methods proposed by Wang B (2021) [49]. Wang B (2021) analyzed the evolution of industrial structure based on psychological laws, stating that the primary industry mainly satisfies humans’ basic survival needs, the secondary industry satisfies humans’ enjoyment needs through industrial products, and the tertiary industry satisfies humans’ advanced needs through services. Therefore, the advanced feature of industrial structure must be that the tertiary industry’s position becomes increasingly prominent, while the proportion of the primary industry’s output value becomes smaller. Thus, in the evaluation of industrial structure advancement, there are differences in weight assignment among industries, with the tertiary industry being assigned the highest weight, the primary industry the lowest, and the secondary industry in the middle. For ease of calculation and understanding, Wang B (2021) assigned a weight of 3 to the tertiary industry, a weight of 2 to the secondary industry, and a weight of 1 to the primary industry. The specific calculation formula is as follows:

$$H=\sum_{i=1}^{3}C_{i}\times i=C_{1}\times 1+C_{2}\times 2+C_{3}\times 3$$ (3)

In Formula (3), H represents the coefficient of industrial structure upgrading, Ci denotes the proportion of output value for the i-th industry, which is equal to Ci/C, where Ci is the output value of the i-th industry, and C is the sum of output values of the three industries.

The coefficient of industrial structure upgrading (H) ranges from 1 to 3. In terms of economic implications, when the coefficient of industrial structure upgrading approaches infinity, the industrial structure of a country or region is at a lower level, indicating a slower pace of optimization. Conversely, when the coefficient of industrial structure upgrading approaches 3, the industrial structure of a country or region is at a higher level, indicating a faster pace of optimization.

#### 4.2.3 Descriptive statistics

According to the evaluation index system for green finance and industrial structure optimization, the levels of green finance and industrial structure optimization in China from 2012 to 2020 were calculated separately. The descriptive statistics of the variables are shown in Table 3.

**Table 3:** Descriptive statistics of the variables.

	Variable	Mean	Std.dev	Min	Max
Explained variables	Advanced industrial structure (H)	63.902	52.094	4.234	280.121
	Rationalization of industrial structure (R)	0.198	0.137	0.008	0.777
Explanatory variables	Green Finance (F)	0.196	0.115	0.071	0.839
Control variables	Technological Progress (T)	15.539	7.328	1.559	29.001
	Human Capital (J)	9.313	0.877	7.679	12.717
	Environmental Regulation (EN)	0.536	0.541	0.001	2.585

**Fig 3:**
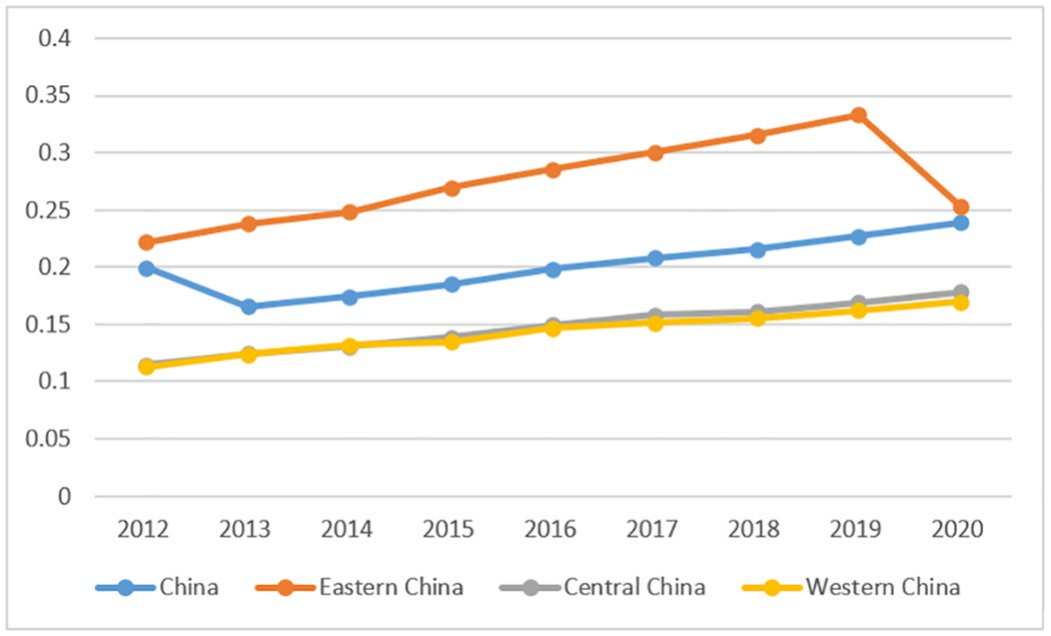
China’s green green finance development level, 2012–2020.

From the perspective of green finance development, the level of green finance development in China has been continuously increasing from 2012 to 2020, as shown in Fig 3. The level of green finance development in China has risen from 0.199 in 2012 to 0.239 in 2020, indicating that China’s green finance system has been continuously improving in recent years, providing strong support for economic activities such as regional environmental improvement, climate change response, and efficient use of resources. In terms of regions, the level of green finance development in China’s eastern region was significantly higher than that in the central and western regions from 2012 to 2020. The level of green finance development in China’s eastern region was higher than the average level of green finance in China, while the level of green finance development in the central and western regions was lower than the average level of green finance in China.

**Fig 4:**
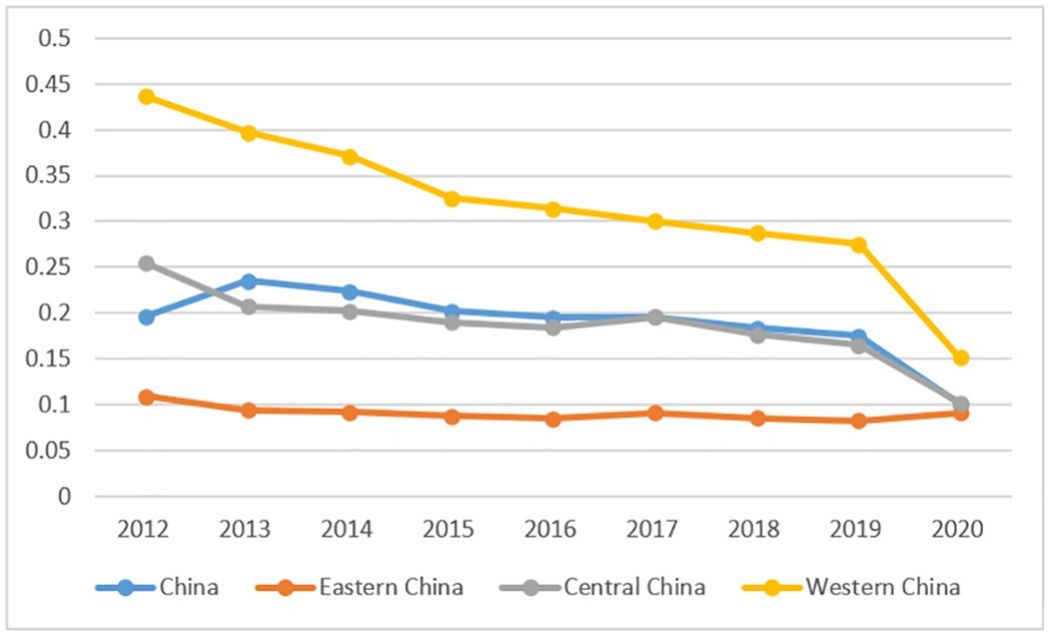
China’s industrial structure rationalization index, 2012–2020.

This article uses the Theil index to measure the degree of industrial structure rationalization during a certain period, mainly based on the consistency between the employment structure and the industrial structure. The closer the industrial structure rationalization index is to 0, the more reasonable the employment structure of the industry is. Conversely, the larger the industrial structure rationalization index, the more unreasonable the structure. The industrial structure rationalization index for each region in China was calculated using Eq (2) as shown in Fig 4. Overall, the industrial structure rationalization index of China from 2012 to 2020 showed a continuous downward trend, but as industrial structure rationalization is a reverse indicator, this indicates that China’s industrial structure is becoming more rational. Looking at the regions, the industrial structure rationalization index in western China is the highest, exceeding the national average, while the index in eastern China is the lowest. The industrial structure rationalization index in central China is basically the same as the national average. This indicates that the level of industrial structure rationalization in eastern China is the highest, while that in western China is the lowest, showing a spatial pattern of industrial structure rationalization in China from east to central to west.

**Fig 5:**
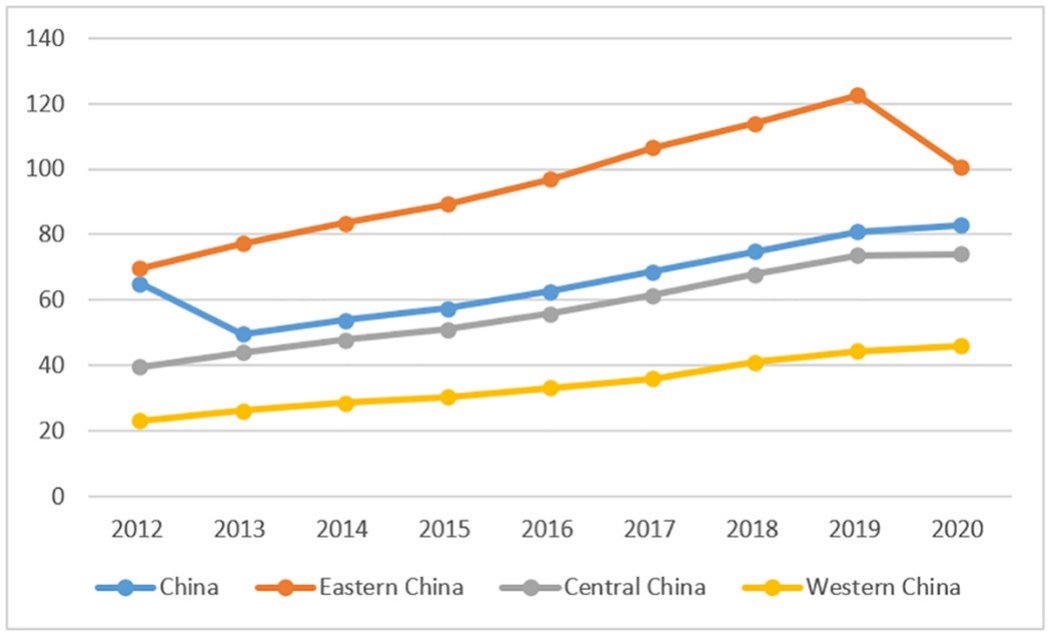
China’s index of advanced industrial structure, 2012–2020.

In terms of the upgrading of industrial structure, the index of China’s industrial structure upgrading showed a continuous upward trend from 65.067 in 2012 to 82.872 in 2020. This indicates that China’s industrial structure has been continuously optimized and the level of upgrading has been constantly improving. According to Chinese statistical data, the proportion of China’s primary industry to GDP fell below 10% in 2010. By 2014, the proportion of China’s tertiary industry to GDP exceeded that of the secondary industry, making the tertiary industry the dominant force in China’s economic development. China has basically achieved a modern industrial structure and formed a stable "3-2-1" industry structure. In terms of regions, the index of industrial structure upgrading in China’s eastern region is the highest, exceeding the national average level. The index in China’s central and western regions is lower than the national average, with the western region having the lowest index of industrial structure upgrading. Since 2012, the spatial pattern of industrial structure upgrading in China’s regions has formed the order of eastern>central>western, as shown in Fig 5.

### 4.3 empirical strategy

This paper first analyzes the overall impact of green finance on the optimization of China’s industrial structure, and then further examines the differences in the effects of green finance on the optimization of China’s industrial structure in different regions and industries from the dimensions of regional heterogeneity and industry heterogeneity.

The impact of green finance on the optimization of regional industrial structure is heterogeneous. Firstly, due to the differences in resource endowments across regions, there are variations in the development level of green finance and the level of industrial structure, resulting in regional heterogeneity in the impact of green finance on industrial structure optimization. Secondly, the differences in the industry attributes and green energy efficiency in the industrial structure result in industry heterogeneity in the effect of green finance on different industries within the same region. This may even lead to completely opposite effects, such as constraining high-energy-consuming industries while promoting green industries, thus indicating the presence of industry heterogeneity.

In terms of regional heterogeneity, the impact of green finance on optimizing industrial structure was analyzed separately for the eastern, central, and western regions of China. In terms of industry heterogeneity, since the impact of green finance on optimizing industrial structure mainly manifests in two aspects: one is the restrictive effect on high-energy-consuming and highly polluting industries; the other is the promotion effect on green industries. Therefore, the differences in the impact of green finance on high-energy-consuming industries and green industries were analyzed separately to verify the role of green finance in optimizing industrial structure.

## 5. Empirical results

### 5.1 Baseline regression analysis

#### 5.1.1 Empirical model

Green finance promotes the rationalization and advanced development of regional industrial structure through capital formation, leverage, industrial integration and other means. Based on this, a econometric model for the optimization of industrial structure by green finance was constructed for hypothesis 1 and hypothesis 2 in this section as follows.


$$LnH_{it}=\alpha_{0}+\alpha_{1}LnF_{it}+\mu_{i}+v_{t}+\varepsilon_{it}$$ (4)

$$LnH_{it}=\alpha_{0}+\alpha_{1}LnF_{it}+\alpha_{2}{\text{control}}_{it}+\mu_{i}+v_{t}+\varepsilon_{it}$$ (5)

$$LnR_{it}=\beta_{0}+\beta_{1}LnF_{it}+\mu_{i}+v_{t}+\varepsilon_{it}$$ (6)

$$LnR_{it}=\beta_{0}+\beta_{1}LnF_{it}+\beta_{2}{\text{control}}_{it}+\mu_{i}+v_{t}+\varepsilon_{it}$$ (7)

In the above equation, t represents time, i represents region, LnH_it_ represents advanced industrial structure in region i in year t, LnR_it_ represents rationalization of industrial structure in region i in year t, LnF_it_ represents green financial development level in region i in year t, control_it_ represents control variables, μ_i_ is individual fixed effect, ν_t_ is time fixed effect, and ε_it_ is random disturbance term. To eliminate the effect of heteroskedasticity, we analyzed the model variables by taking logarithms.

#### 5.1.2 Variable descriptions

*1*. *Explained variables*. This article uses the advanced and rationalized industrial structure as measures to evaluate the optimization of regional industrial structure. Therefore, the explanatory variables include two variables: the advanced industrial structure (H) as a positive indicator, and the rationalized industrial structure (R) as a negative indicator.

*2*. *Explanatory variables*. We use the entropy-weighted TOPSIS method to calculate the level of green finance (F) development in each province of China from four aspects: green credit, green investment, green insurance, and green fiscal spending.

*3*. *Control variables*. Referring to the existing literature [50–52], we selects total factor productivity (T), human capital (C), and environmental regulation (E) as control variables.

Total Factor Productivity (T) can promote both the upgrade and optimization of economic structure, and the changes and adjustments in industrial structure. Firstly, T can promote the growth of overall economic productivity and technological progress, which will drive the transfer of resources from low-efficiency sectors to high-efficiency sectors, thereby affecting industrial structure. Secondly, T will promote innovation and the development of new technologies, improve the competitiveness of enterprises, and change the industrial structure. Thirdly, differences in T will affect the competitiveness and survival of different industries and enterprises, which will affect the evolution of industrial structure [53].

Human capital (C) refers to an individual’s abilities in terms of education, skills, health, experience, and knowledge, among other aspects. The increase of human capital can enhance an individual’s labor productivity and creativity, which in turn affects the industrial structure. Firstly, the increase of human capital can enhance the competitiveness of certain industries and enterprises, making them more capable of development and thereby changing the industrial structure. Secondly, high levels of human capital contribute to innovation and the development of new technologies, thereby changing the industrial structure. Thirdly, the increase of human capital can improve the labor quality and productivity of certain industries, promoting the adjustment and optimization of the industrial structure [54].

Environmental regulation (E). Firstly, environmental regulation will increase the environmental protection costs for enterprises, which will exert certain pressures on their operation. This may lead to the exit of some high-pollution and high-energy-consuming industries from the market, while some industries with better environmental performance will have more development opportunities, thus changing the industrial structure. Secondly, environmental regulation helps promote the upgrade and optimization of industrial technology, making enterprises with more environmentally friendly technology more competitive, while those that do not meet environmental requirements face greater survival pressure. This may lead to changes and adjustments in industrial structure. Thirdly, environmental regulation can promote the development of more environmentally friendly industries such as new energy and clean technology. These industries often have higher technical barriers and investment costs, but under environmental pressure, the government and the market will also support the development of these industries more, thus changing the industrial structure [13].

The specific explanatory variables, explanatory variables, and control variables indicators are described in Table 4.

**Table 4:** Description of variable indicators.

	Variables	Calculation method	Indicator Properties
Explained variables	Advanced industrial structure (H)	Calculated from equation (4.14)	Positive indicators
	Rationalization of industrial structure (R)	Calculated from equation (4.13)	Negative indicators
Explanatory variables	Green Finance (F)	Calculated from equation (4.11)	Positive indicators
Control variables	Technological Progress (T)	Calculated from the C-D function	Positive indicators
	Human Capital (J)	Years of education per capita (years)	Positive indicators
	Environmental Regulation (EN)	Industrial pollution control completed investment (million yuan) / secondary industry added value (million yuan)	Positive indicators

#### 5.1.3 Analysis of regression results

By conducting a Hausman test, it was found that the statistic value is 9.76 and the corresponding p-value is 0.002. This means that the null hypothesis of the presence of random effects can be rejected. Therefore, using a fixed effects panel model would result in better analysis outcomes. Table 5 reports the results of the benchmark estimates of the impact of green finance on the advanced industrial structure and the rationalization of industrial structure in China over the period 2012–2020.

**Table 5:** Baseline regression estimation results.

	(1)	(2)	(3)	(4)
	LNR	LNH	LNR	LNH
LNF	-1.421***	1.370***	-1.275***	1.229***
	(0.1130)	(0.0510)	(0.1589)	(0.0729)
LNT			0.109***	0.0124
			(0.0258)	(0.0118)
LNC			-0.688	0.960*
			(1.0417)	(0.4779)
LNE			-0.108*	0.0620**
			(0.0419)	(0.0192)
_cons	-1.904***	2.700***	-1.192	1.618**
	(0.0857)	(0.0387)	(1.0940)	(0.5019)
Temporal Fixed Effect	YES	YES	YES	YES
Regional Fixed Effect	YES	YES	YES	YES
*N*	270	270	270	270
*R* ^2^	0.3985	0.7514	0.4550	0.7671

Standard errors in parentheses

* p < 0.05,

** p < 0.01,

*** p < 0.001

The impact of green finance on the rationalization of industrial structure. Column (1) estimates the negative regression coefficient of green finance on industrial structure rationalization without the inclusion of control variables. However, since industrial structure rationalization is a negative indicator, green finance has a significant contribution to industrial structure rationalization. Column (3) further introduces control variables based on this and controls for individual effects and time effects. It was found that the sign of the core explanatory variables did not change and the goodness-of-fit value of the model increased from 0.3985 to 0.4550. This indicates that the selection of control variables in this paper is reasonable and the baseline estimation results are more stable. From the regression results in column (3), we can get that at the significance level of 1‰, green finance has a significant contribution to the rationalization of industrial structure, and every 1 percentage point increase in green finance will promote the growth of industrial structure rationalization by 1.275 percentage points. Accordingly, hypothesis 1 is verified.

The impact of green finance on the advanced industrial structure. Column (2) estimates the effect of green finance on the advanced industrial structure without the inclusion of control variables. The regression coefficient of green finance on the advanced industrial structure is positive. Since the advanced industrial structure is a positive indicator, green finance has a significant contribution to the advanced industrial structure. Column (4) further introduces control variables on this basis and controls for both individual effects and time effects. We can find that the sign of the core explanatory variables does not change, and the regression coefficients improve significantly, and the goodness-of-fit value of the model increases from 0.7514 to 0.7671. This indicates that the selection of the control variables in this paper is reasonable and the baseline estimation results are more stable. From the regression results in column (4), it shows that green finance has a significant contribution to the advanced industrial structure at a significance level of 1‰, and each 1 percentage point increase in green finance will promote 1.229 percentage points of growth in the advanced industrial structure. Accordingly, hypothesis 2 is verified.

Considering that there may be some lags in the effects of green finance policy implementation, we robustly test the model by lagging the core explanatory variables and control variables by one period simultaneously. The robustness test is estimated by estimating the core explanatory and control variables in the previous period with the explanatory variables in the current period, and the estimation results are shown in Table 6. The robustness test results show that both the sign and significance level of the estimated coefficients of the explanatory variables on the explained variables are generally consistent with the regression results in the corresponding models, which indicates that the baseline regression results have a high degree of confidence.

**Table 6:** Robustness tests.

	(1)	(2)	(3)	(4)
	LNR	LNH	LNR	LNH
LNF	-0.817***	1.457***	-0.766***	1.259***
	(0.0789)	(0.0631)	(0.1072)	(0.0828)
LNT			0.0209	0.00610
			(0.0170)	(0.0131)
LNC			-0.477	1.436**
			(0.6245)	(0.4821)
LNE			-0.0369	0.0600**
			(0.0258)	(0.0199)
cons	-1.400***	2.734***	-0.906	1.161*
	(0.0589)	(0.0471)	(0.6565)	(0.5069)
Temporal Fixed Effect	YES	YES	YES	YES
Regional Fixed Effect	YES	YES	YES	YES
*N*	240	240	240	240
*R* ^2^	0.3389	0.7183	0.3504	0.7420

Standard errors in parentheses

* p < 0.05,

** p < 0.01,

*** p < 0.001

### 5.2 Regional heterogeneity analysis

Due to the difference of resource endowment in each region, there is variability in the level of green finance development and industrial structure level in each region, so there is regional heterogeneity in the optimization of industrial structure by green finance. Before conducting regression analysis with panel data, it is necessary to use the Hausman test to determine the type of panel data model. Based on the Hausman test, the statistic value is 8.26 and the corresponding p-value is 0.004, indicating that the null hypothesis of the presence of random effects can be rejected. Therefore, using a fixed effects spatial panel model would result in better analysis outcomes.

The regression analysis reveals that the development of green finance in all regions of China significantly contributes to the rationalization and advanced regional industrial structure at a significance level of 1‰, but the degree of its impact is regionally heterogeneous among eastern China, central China and western China. The effect of green finance on the optimization of industrial structure is higher in central and western China than in eastern China. As shown in Table 7. The reason for this is that the industrial structure in eastern China is higher than that in central and western China, and there are still more high energy-consuming and high-polluting enterprises in central and western China, while a modern industrial system with service industry as the mainstay has been formed in eastern China. Therefore, the development of green finance has a less constraining effect on the optimization of industrial structure in eastern China, so the impact of green finance on China’s industrial structure shows a spatial pattern of western China > central China > eastern China. Accordingly, hypothesis 3 is verified.

**Table 7:** Regression results of regional heterogeneity.

	Western China				Central China				Eastern China			
	(1)	(2)	(3)	(4)	(5)	(6)	(7)	(8)	(9)	(10)	(11)	(12)
	LNR	LNH	LNR	LNH	LNR	LNH	LNR	LNH	LNR	LNH	LNR	LNH
LNF	-1.868***	1.621***	-1.794***	1.513***	-1.365***	1.317***	-1.251***	1.207***	-1.121***	1.216***	-0.965**	1.038***
	(0.1854)	(0.0837)	(0.2605)	(0.1222)	(0.2058)	(0.0938)	(0.2797)	(0.1360)	(0.1896)	(0.0833)	(0.3131)	(0.1343)
LNT			0.123**	0.0007			0.124*	0.0229			0.0929*	0.0123
			(0.0415)	(0.0194)			(0.0554)	(0.0269)			(0.0447)	(0.0192)
LNC			0.332	0.411			-0.217	1.099			-1.231	1.353
			(1.3306)	(0.6242)			(2.1691)	(1.0546)			(2.3965)	(1.0278)
LNE			-0.132	0.0605			-0.136	0.0353			-0.0692	0.0783**
			(0.0891)	(0.0418)			(0.0752)	(0.0366)			(0.0663)	(0.0284)
cons	-2.150***	2.819***	-2.494	2.316***	-1.919***	2.815***	-1.687	1.617	-1.820***	2.592***	-0.556	1.069
	(0.1602)	(0.0723)	(1.4104)	(0.6616)	(0.1732)	(0.0789)	(2.2713)	(1.1043)	(0.1125)	(0.0494)	(2.5209)	(1.0811)
Temporal Fixed Effect	YES	YES	YES	YES	YES	YES	YES	YES	YES	YES	YES	YES
Regional Fixed Effect	YES	YES	YES	YES	YES	YES	YES	YES	YES	YES	YES	YES
*N*	99	99	99	99	72	72	72	72	108	108	108	108
*R* ^2^	0.5384	0.8115	0.5858	0.8176	0.4112	0.7579	0.5023	0.7672		0.7103	0.2868	0.7405

Standard errors in parentheses

* p < 0.05,

** p < 0.01,

*** p < 0.001

By region, columns (1)-(4) estimate the impact of green finance development on the regional industrial structure in western China. The sign of the explanatory variables did not change based on the introduction of control variables and controlling for both individual and time effects. This indicates that the selection of control variables in this paper is reasonable and the baseline estimation results are more stable. The regression results in column (3) indicate that at a significance level of 1‰, green finance in western China has a significant contribution to the rationalization of industrial structure, and each 1 percentage point increase in green finance will contribute to a 1.794 percentage point increase in the rationalization of industrial structure. The regression results in column (4) indicate that at a significance level of 1‰, green finance in western China has a significant contribution to the advanced industrial structure, and a 1 percentage point increase in green finance will contribute to a 1.513 percentage point increase in the rationalization of the industrial structure.

Columns (5)-(8) estimate the impact of green finance development on the regional industrial structure in central China. The sign of the explanatory variables did not change based on the introduction of control variables and controlling for both individual and time effects. This indicates that the selection of control variables in this paper is reasonable and the baseline estimation results are relatively stable. The regression results in column (7) show that at the significance level of 1‰, green finance in central China has a significant contribution to the rationalization of industrial structure, and each 1 percentage point increase in green finance will promote the rationalization of industrial structure by 1.271 percentage points. The regression results in column (8) show that at the significance level of 1‰, green finance in central China has a significant contribution to the advanced industrial structure, and each 1 percentage point increase in green finance will promote the growth of industrial structure rationalization by 1.207 percentage points.

Columns (9)-(12) estimate the impact of green finance development on the regional industrial structure in eastern China. The sign of the explanatory variables did not change based on the introduction of control variables and controlling for both individual and time effects. This indicates that the selection of control variables in this paper is reasonable and the baseline estimation results are relatively stable. The regression results in column (11) show that green finance has a significant contribution to industrial structure rationalization in eastern China at a significance level of 1‰. Each 1 percentage point increase in green finance will promote 0.965 percentage points of growth in industrial structure rationalization. The regression results in column (12) show that green finance in eastern China has a significant contribution to the advanced industrial structure at a significance level of 1‰. Each 1 percentage point increase in green finance will promote 1.038 percentage points of growth in industrial structure rationalization.

### 5.3 Industry heterogeneity analysis

The effect of green finance on the optimization of industrial structure is mainly reflected in the differential allocation of funds to different industries.

Through the development of green finance, capital can be directed to industries with low pollution and high resource and energy utilization. This is conducive to encouraging the energy-saving and environmental protection industry to develop and promote new technologies and products, forcing high-energy-consuming enterprises to carry out technological transformation and realize the upgrading of traditional industries. This will reduce the consumption of resources and energy and damage to the natural environment, and promote the overall green transformation of economic and social development. Based on this, this section analyzes the impact of green finance on industrial structure by industry.

#### 5.3.1 Empirical model construction

Since there is a differential impact of green finance on green industries and high-energy industries. To further verify the mechanism of the effect of green finance on industrial structure optimization. This study constructs the econometric models of green finance on green industry and high energy consumption industry in China separately as follows.


$$LnGRE_{it}=\alpha_{0}+\alpha_{1}LnF_{it}+\mu_{i}+v_{t}+\varepsilon_{it}$$ (8)

$$LnGRE_{it}=\alpha_{0}+\alpha_{1}LnF_{it}+\alpha_{2}{\text{control}}_{it}+\mu_{i}+v_{t}+\varepsilon_{it}$$ (9)

$$LnHE_{it}=\beta_{0}+\beta_{1}LnF_{it}+\mu_{i}+v_{t}+\varepsilon_{it}$$ (10)

$$LnHE_{it}=\beta_{0}+\beta_{1}LnF_{it}+\beta_{2}{\text{control}}_{it}+\mu_{i}+v_{t}+\varepsilon_{it}$$ (11)

In the above equation, t represents time, i represents region, LnGRE_it_ represents the development level of green industry in region i in year t, LnHE_it_ represents the development level of high energy consumption industry in region i in year t, and LnF_it_ represents green finance in region i in year t. Control_it_ represents control variables, and the selection of control variables is consistent with the above, choosing total factor productivity (T), human capital (C), and environmental regulation (E) as control variables.μ_i_ is the individual fixed effect, ν_t_ is the time fixed effect, and ε_it_ is the random disturbance term. To eliminate the effect of heteroskedasticity in the model data, the model variables are taken as logarithms. The study sample period is 2012–2020.

Also, to further analyze the impact of green finance on each industry within the green industry and the high-energy industry. We analyze the green industry and the high energy consumption industry separately.

In terms of green industries, this study specifies the green industries into three major industries: agriculture and food processing industry, pharmaceutical manufacturing industry, and computer, communication and other electronic equipment manufacturing industry. The impact model of green finance on each industry in the green industry is as follows.


$$LnMED_{it}=\alpha_{0}+\alpha_{1}LnF_{it}+\alpha_{2}{\text{control}}_{it}+\mu_{i}+v_{t}+\varepsilon_{it}$$ (12)

$$LnCOM_{it}=\alpha_{0}+\alpha_{1}LnF_{it}+\alpha_{2}{\text{control}}_{it}+\mu_{i}+v_{t}+\varepsilon_{it}$$ (13)

$$LnARG_{it}=\alpha_{0}+\alpha_{1}LnF_{it}+\alpha_{2}{\text{control}}_{it}+\mu_{i}+v_{t}+\varepsilon_{it}$$ (14)

In the above equation, t represents time, i represents region, LnMED_it_ represents the development level of pharmaceutical manufacturing industry in region in year i, LnCOM_it_ represents the development level of computer, communication and other electronic equipment manufacturing industry in region in year i, LnARG_it_ represents the development level of agriculture and food processing industry in region in year i, and LnF_it_ represents green finance in region in year i. Control_it_ represents control variables, and total factor productivity (T), human capital (C) and environmental regulation (E) are selected as control variables. μ_i_ is an individual fixed effect, ν_t_ is a time fixed effect, and ε_it_ is a random disturbance term. To eliminate the effect of heteroskedasticity in the data, the model variables are logarithmic. The study sample period is 2012–2020.

In terms of high-energy-consuming industries, according to the definition of six high-energy-consuming industries in the 2016 National Economic and Social Development Statistical Bulletin. The high-energy-consuming industries are limited to six industries: petroleum, coal and other fuel processing industry, chemical raw materials and chemical products manufacturing industry, production and supply of electricity and heat, non-metallic mineral products industry, ferrous metal smelting and rolling processing industry, and non-ferrous metal smelting and rolling processing industry. In order to verify whether green finance has a suppressive effect on the development of high energy-consuming industries in China, we constructed a model of the impact of green finance on each industry of high energy-consuming industries. The model is as follows.


$$LnOIL_{it}=\beta_{0}+\beta_{1}LnF_{it}+\beta_{2}{\text{control}}_{it}+\mu_{i}+v_{t}+\varepsilon_{it}$$ (15)

$$LnCHE_{it}=\beta_{0}+\beta_{1}LnF_{it}+\beta_{2}{\text{control}}_{it}+\mu_{i}+v_{t}+\varepsilon_{it}$$ (16)

$$LnELE_{it}=\beta_{0}+\beta_{1}LnF_{it}+\beta_{2}{\text{control}}_{it}+\mu_{i}+v_{t}+\varepsilon_{it}$$ (17)

$$LnMET_{it}=\beta_{0}+\beta_{1}LnF_{it}+\beta_{2}{\text{control}}_{it}+\mu_{i}+v_{t}+\varepsilon_{it}$$ (18)

$$LnBLA_{it}=\beta_{0}+\beta_{1}LnF_{it}+\beta_{2}{\text{control}}_{it}+\mu_{i}+v_{t}+\varepsilon_{it}$$ (19)

$$LnCOL_{it}=\beta_{0}+\beta_{1}LnF_{it}+\beta_{2}{\text{control}}_{it}+\mu_{i}+v_{t}+\varepsilon_{it}$$ (20)

In the above equation, t represents time, i represents region, LnOIL_it_ represents the development level of petroleum, coal and other fuel processing industry in region i in year t, LnCHE_it_ represents the development level of chemical raw materials and chemical products manufacturing industry in region i in year t, LnELE_it_ represents the development level of electric power and heat production and supply industry in region i in year t, LnMET_it_ represents the development level of non-metallic mineral products industry in region i in year t, LnBLA_it_ represents the development level of ferrous metal smelting and rolling processing industry in year t, LnCOL_it_ represents the development level of nonferrous metal smelting and rolling processing industry in year t, and LnF_it_ represents green finance in year t. Control_it_ represents the control variables, and total factor productivity (T), human capital (C), and environmental regulation (E) are selected as control variables. μ_i_ is the individual fixed effect, ν_t_ is the time fixed effect, and ε_it_ is the random disturbance term. To eliminate the effect of heteroskedasticity in the data, the model variables are taken for logit analysis. The study sample period is 2012–2020.

#### 5.3.2 Empirical regression analysis

*1*.*Analysis of industry heterogeneity of green finance*. Before conducting regression analysis with panel data, it is necessary to use the Hausman test to determine the type of panel data model. Based on the Hausman test, the statistic value is 7.68 and the corresponding p-value is 0.001, indicating that the null hypothesis of the presence of random effects can be rejected. Therefore, using a fixed effects spatial panel model would result in better analysis outcomes. Table 8 reports the estimated results of the impact of green finance on green and energy-intensive industries in China over the period 2012–2020.

**Table 8:** Results of industry heterogeneity regression analysis.

	(1)	(2)	(3)	(4)
	LnGRE	LnHE	LnGRE	LnHE
LNF	65.35***	-127.9***	65.54***	-153.4***
	(5.1910)	(14.5796)	(11.8236)	(20.2807)
LNT			0.773	-17.05***
			(2.7641)	(3.2943)
LNC			-1.802	266.9*
			(58.6457)	(132.9873)
LNE			-15.49***	-7.186
			(4.2292)	(5.3501)
cons	87.30***	-37.82***	96.65	-292.2*
	(4.7553)	(11.0598)	(64.5408)	(139.6599)
Temporal Fixed Effect	YES	YES	YES	YES
Regional Fixed Effect	YES	YES	YES	YES
N	270	270	270	270
*R* ^2^	0.2178	0.2435	0.2361	0.3293

Standard errors in parentheses

* p < 0.05,

** p < 0.01,

*** p < 0.001

In terms of the impact of green finance on green industries. Column (1) estimates that the regression coefficient of green finance on green industry is positive with a significance level of 1‰ when control variables are not included. This indicates that green finance has a significant contribution to the green industry. Column (3) further introduces control variables on this basis and controls for individual and time effects, the sign of the explanatory variables does not change, the regression coefficient is significantly improved, and the goodness-of-fit value of the model increases from 0.2178 to 0.2361. This indicates that the choice of control variables in this paper is reasonable and the baseline estimation results are relatively stable. The regression results in column (3) show that green finance makes a significant contribution to the development of green industries in China at the significance level of 1 per 1,000. Each 1 percentage point increase in green finance will contribute 65.54 percentage points to the growth of green industry. Accordingly, hypothesis 4 is verified.

In terms of the impact of green finance on high energy-consuming industries. Column (2) estimates the effect of green finance on high energy consuming industries and the regression coefficient of green finance on high energy consuming industries is negative at 1% significance level without adding control variables. It indicates that green finance has a significant inhibitory effect on high energy-consuming industries. Column (4) further introduces control variables on this basis, while controlling for individual effects and time effects, the sign of the explanatory variables does not change, the regression coefficients are significantly improved, and the goodness-of-fit value of the model increases from 0.2435 to 0.3293. This indicates that the selection of control variables in this paper is reasonable and the baseline estimation results are relatively stable. The regression results in column (4) show that green finance has a significant inhibitory effect on the development of energy-intensive industries in China at the significance level of 1 per 1,000. Each 1 percentage point increase in green finance will lead to a 153.4 percentage point decrease in energy-intensive industries. Accordingly, hypothesis 5 is verified.

To further analyze the impact of green finance on various industries within the green and energy-intensive industries, further analysis by industry is presented below.

*2*. *Analysis of the impact of green finance on green industry*. Before conducting regression analysis with panel data, it is necessary to use the Hausman test to determine the type of panel data model. Based on the Hausman test, the statistic value is 6.24 and the corresponding p-value is 0.001, indicating that the null hypothesis of the presence of random effects can be rejected. Therefore, using a fixed effects spatial panel model would result in better analysis outcomes. Table 9 reports the estimated results of the impact of green finance on China’s green industry over the period 2012–2020.

**Table 9:** Regression estimation results of green finance on green industry.

	(1)	(2)	(3)
	LnMED	LnCOM	LnARG
LNF	10.95***	55.06***	1.404***
	(1.0630)	(11.5379)	(0.2373)
LNT	-0.0353	0.926	0.0313
	(0.2485)	(2.7337)	(0.0398)
LNC	-6.041	3.760	0.199
	(5.2724)	(57.2928)	(1.4658)
LNE	-0.977*	-14.49***	0.0219
	(0.3802)	(4.1045)	(0.0542)
cons	21.85***	75.75	1.409
	(5.8024)	(63.0198)	(1.5290)
Temporal Fixed Effect	YES	YES	YES
Regional Fixed Effect	YES	YES	YES
*N*	270	270	270
*R* ^2^	0.4487	0.1973	0.3205

Standard errors in parentheses

* p < 0.05,

** p < 0.01,

*** p < 0.001

By industry, column (1) estimates the impact of green finance on pharmaceutical manufacturing industry, and the regression coefficient of green finance on pharmaceutical manufacturing industry is positive at the significance level of 1‰. It indicates that green finance has a significant promotion effect on pharmaceutical manufacturing industry, and every 1 percentage point increase in green finance will promote 10.95 percentage points of growth in pharmaceutical manufacturing industry.

Column (2) estimates the effect of green finance on communication and other electronic equipment manufacturing industry. The regression coefficient of green finance on communication and other electronic equipment manufacturing industry is positive at the significance level of 1‰. It indicates that green finance has a significant contribution to the communication and other electronic equipment manufacturing industry, and each 1 percentage point increase in green finance will promote 55.06 percentage points of growth in the communication and other electronic equipment manufacturing industry.

Column (3) estimates the impact of green finance on agri-food processing industry, and the regression coefficient of green finance on agri-food processing industry is positive at the significance level of 1‰. It indicates that green finance has a significant promotion effect on the agrifood processing industry, and every 1 percentage point increase in green finance will promote 1.404 percentage points of growth in the agrifood processing industry.

Overall, green finance has a significant positive contribution to the development of green industries in China. Among them, green finance makes the largest contribution to the communication and other electronic equipment manufacturing industry. This indicates that greening and digitization have become important drivers for the current high-quality development of China’s economy.

*3*. *Analysis of the impact of green finance on energy-intensive industries*. Based on the Hausman test, it is known that the statistic value is 7.35 and the corresponding p-value is 0.003. This means that the null hypothesis of the presence of random effects can be rejected. Therefore, using a fixed effects spatial panel model would result in better analysis outcomes. Table 10 reports the estimated results of the impact of green finance on China’s energy-intensive industries over the period 2012–2020.

**Table 10:** Regression estimation results of green finance for energy-intensive industries.

	(1)	(2)	(3)	(4)	(5)	(6)
	LnCHE	LnOIL	LnMET	LnBLA	LnCOL	LnELE
LNF	-34.20***	-2.627***	-28.02***	-40.66***	-7.278***	-6.091**
	(5.3704)	(0.6740)	(5.5419)	(4.3060)	(1.1103)	(2.0321)
LNT	-0.920	-0.149	-1.487	-1.783*	-0.362*	-0.451
	(0.8723)	(0.1095)	(0.9002)	(0.6994)	(0.1804)	(0.3301)
LNC	68.77	-3.908	75.81*	48.02	2.674	32.62*
	(35.2153)	(4.4197)	(36.3399)	(28.2361)	(7.2809)	(13.3254)
LNE	-3.471*	0.257	-2.308	-1.325	-0.753*	-1.101*
	(1.4167)	(0.1778)	(1.4620)	(1.1359)	(0.2929)	(0.5361)
cons	-75.02*	4.776	-73.76	-63.81*	-0.962	-25.79
	(36.9822)	(4.6415)	(38.1632)	(29.6528)	(7.6462)	(13.9940)
Temporal Fixed Effect	YES	YES	YES	YES	YES	YES
Regional Fixed Effect	YES	YES	YES	YES	YES	YES
*N*	270	270	270	270	270	270
*R* ^2^	0.2354	0.1593	0.1454	0.4066	0.3118	0.0665

Standard errors in parentheses

* p < 0.05,

** p < 0.01,

*** p < 0.001

Columns (1)-(6) estimate the impact of green finance on each of the six energy-intensive industries in China. With the introduction of control variables and controlling for individual and time effects, the effects of green finance on the six energy-intensive industries in China are all negative at the 1‰ significance level. This suggests that green finance has a dampening effect on China’s energy-intensive industries.

By industry, green finance has the greatest inhibitory effect on the ferrous metal smelting and rolling processing industry, with each 1 percentage point increase in green finance reducing the ferrous metal smelting and rolling processing industry by 40.66 percentage points. Green finance on chemical raw materials and chemical products manufacturing, non-metallic mineral products industry also has a greater inhibitory effect. Each 1 percentage point increase in green finance will lead to a 34.20 percentage point decrease in the chemical raw materials and chemical products manufacturing industry. Each 1 percentage point increase in green finance will lead to a decline of 28.02 percentage points in the non-metallic mineral products industry. Green finance on petroleum, coal and other fuel processing industry, electricity, heat production and supply industry, non-ferrous metal smelting and rolling processing industry has a smaller impact. Each 1 percentage point increase in green finance will result in a 2.627 percentage point decrease in the petroleum, coal and other fuel processing industry. Each 1 percentage point increase in green finance will result in a 6.091 percentage point decrease in the production and supply of electricity and heat. Each 1 percentage point increase in green finance will result in a 7.278 percentage point decrease in the smelting and rolling processing of non-ferrous metals industry.

## 6. Conclusion and policy implications

### 6.1 Conclusion

#### 1. Green finance is an important driving force for promoting industrial structure optimization

From a theoretical mechanism analysis perspective, as a financial service activity that supports the development of green economy and the transformation of economic greening, green finance is of great significance for industrial transformation and upgrading. Green finance can promote the optimization of industrial structure through fund guidance mechanism, information transmission mechanism, and resource integration mechanism. From the perspective of empirical analysis results, at the significant level of 1‰, the development of green finance has a significant promoting effect on the rationalization and high-level of industrial structure.

#### 2. China’s green finance and industrial structure optimization still exist imbalances and insufficiencies

From the perspective of green finance development, the level of green finance development in China from 2012 to 2020 has been on the rise. In terms of regions, the level of green finance development in the eastern region of China was significantly higher than that in the central and western regions from 2012 to 2020. The level of green finance development in the eastern region of China was higher than the average level of green finance in China, while the level of green finance development in the central and western regions was lower than the average level of green finance in China. In terms of optimizing industrial structure, the industrial structure in China has been tending towards rationalization from 2012 to 2020. In terms of regions, the level of industrial structure rationalization in the eastern region of China was the highest, while that in the western region was the lowest, presenting a spatial pattern of industrial structure rationalization from east to central to west in China. In terms of upgrading industrial structure, the industrial structure in China has been continuously optimized and upgraded from 2012 to 2020. In terms of regions, the index of upgrading industrial structure in the eastern region of China was the highest, which was higher than the national average level. The index of upgrading industrial structure in the central and western regions of China was lower than the national average level, with the lowest index in the western region of China.

#### 3. The impact of green finance on industrial structure optimization exhibits heterogeneity

From the perspective of empirical analysis results, the impact of green finance on the optimization of China’s industrial structure exhibits obvious heterogeneity. In terms of regional heterogeneity, at the significance level of 1‰, the effect of green finance on the optimization of the industrial structure in the central and western regions of China is higher than that in the eastern region, and the impact of green finance on China’s industrial structure shows a spatial pattern of western China > central China > eastern China. In terms of industry heterogeneity, at the significance level of 1‰, green finance has a significant promoting effect on the development of green industries, and a significant inhibiting effect on the development of high-energy-consuming industries. Specifically, in terms of green industries, green finance has the greatest promoting effect on the communication and other electronic equipment manufacturing industry; in terms of high-energy-consuming industries, the greatest inhibiting effect of green finance is on the black metal smelting and rolling processing industry, and the smallest impact is on the petroleum, coal and other fuel processing industries.

### 6.2 Policy implications

#### 6.2.1 Implications for the government

*1*. *Strive to create a favorable financial ecosystem*. The government can guide the healthy development of green finance institutions and encourage the green industrialization of related enterprises through the formulation of relevant policies, using green credit as a lever to promote the transformation of economic development mode and adjustment of industrial structure. In addition, incentive mechanisms such as establishing national and regional green development funds by integrating existing special funds for energy conservation and environmental protection, and implementing financial interest subsidies and tax exemptions for green environmental projects and enterprises can also be established.

*2*. *Vigorously promote the construction of green finance infrastructure*. Push for the standardization of green finance, guide all types of market entities to accurately grasp policy guidance, and accelerate the green upgrading of the domestic economy; promote the construction of a green credit system, establish a credit information sharing platform, and create a credit information sharing mechanism. Improve the effective disclosure of green information, require strengthened information exchange among environmental protection, regulatory authorities, and banking and financial institutions, and include information on environmental violations, project environmental impact assessments, environmental protection inspections and mandatory clean production audits by enterprises into the People’s Bank of China’s credit reporting system and the financial credit information basic database. Establish and improve the mandatory environmental information disclosure system for relevant enterprises, and strive to explore the establishment of third-party assessment and rating standards, and cultivate and standardize the ability and evaluation quality requirements of third-party agencies to provide environmental information disclosure services to green enterprises.

*3*. *Actively explore the establishment of a green finance monitoring system*. Financial regulatory authorities should strengthen the supervision of the implementation of green development by financial institutions and companies. They should not only include the development of green finance in their daily supervision, promptly obtain first-hand information, but also actively conduct investigation and research, clarify the experience and problems of green finance development, and lay the foundation for the next step of green work. Although the green finance market has a risk redistribution function, the establishment of monitoring systems can reduce the probability of unavoidable risks during the implementation of green finance and actively prevent and control "two highs and one remaining" risks and further strengthen risk compensation mechanisms.

#### 6.2.2 Implications for financial institutions

*1*. *Strictly implementing differential interest rate policy*. Banking and financial institutions use means such as interest rate incentives to significantly increase credit allocation in green production and consumption fields, implementing positive incentives such as preferential interest rates for environmentally compliant and green, pollution-free companies, and ecological agriculture enterprises. High interest rates are implemented for industries that do not meet environmental requirements, which encourages companies to increase environmental investment and enhance environmental protection efforts. Through differentiated policies, the "two highs and one surplus" industries are transformed, eliminated, and exited to a certain extent.

*2*. *Enhance the innovation capability of green finance*. The main tool and means of green finance is green credit. Although green credit has shown steady growth in scale, significant environmental benefits, overall good credit quality, and low non-performing loan ratio, financial institutions still need to vigorously develop green finance business and continuously increase the innovation and vitality of green finance. In the process of innovating green finance products and services, financial institutions should integrate environmental and social responsibility concepts on the basis of learning from successful experiences of traditional financial products and services, while taking into account both economic and social benefits, providing more effective financial services for the development of green and environmental protection enterprises and the transformation of polluting enterprises. For example, emissions trading mortgage loans can not only be used for enterprise energy-saving and emission reduction technology transformation to promote energy-saving, emission reduction, and transformation and upgrading but also can use loan funds to purchase emissions trading rights, expanding the use of emissions trading mortgage loans, which is positively significant for revitalizing the emissions trading market and promoting emissions trading. Green bonds can actively broaden the channels for direct financing of green finance, effectively reduce financing thresholds, and leverage diversified social capital to invest in the green industry at a lower financing cost and longer return period. Safety and environmental liability insurance, through insurance integration, government and insurance cooperation, and the introduction of third-party safety and environmental protection service organizations, can effectively improve environmental management, reduce business risks, and reduce excessive emissions.

## 7. Future research directions and limitations

Firstly, it is necessary to improve the accuracy of measuring indicators for the development level of green finance. Currently, the development of green finance in China is in its initial stage, and the statistics and disclosure of related data are still being improved. The use of alternative measurement indicators may result in deviations from the actual situation, which has some impact on the measurement of the development level of green finance. With the continuous development of green finance and the improvement of data, it is possible to construct a more comprehensive and scientific indicator system to obtain more accurate conclusions.

Secondly, it is important to explore the mediating pathways of green finance on the optimization of industrial structure. This study focuses on the "green finance-resource allocation-industrial structure optimization" pathway, and future research can examine other possible mediating variables through a combination of theory and empirical analysis, such as the "green finance-green technology-industrial structure optimization" pathway.

Thirdly, further research can be conducted on industrial heterogeneity. Due to data availability, this study investigates the industry heterogeneity of high-pollution and high-energy-consuming industries and green industries from a macro perspective. In the future, more targeted and constructive development recommendations can be proposed for different industries through further research on manufacturing, services, and other sectors.

## Supporting information

S1 Data (XLS)
